# Perceived surgical difficulty of mandibular third molar extraction. A comparative cross-sectional study of dentists with postgraduate qualification in oral surgery and maxillofacial surgeons in a Spanish subpopulation

**DOI:** 10.4317/medoral.26243

**Published:** 2024-01-30

**Authors:** María Isabel Sánchez-Jorge, Jorge Cortés-Bretón-Brinkmann, Rosa Acevedo-Ocaña, Norberto Quispe-López, Farzin Falahat, Rafael Martín-Granizo

**Affiliations:** 1 Department of Oral Surgery and Oral Implantology, Faculty of dentistry, University Alfonso X el Sabio, Madrid, Spain; 2 Department of Dental Clinical Specialties, Faculty of dentistry, Complutense University of Madrid, Spain; 3 Surgical and Implant Therapies in the Oral Cavity Research Group, University Complutense, Madrid, Spain; 4 Department of Surgery, Faculty of Medicine, University of Salamanca, Spain; 5 Faculty of Medicine, Complutense University of Madrid, Spain; 6 Department of Oral and Maxillofacial Surgery, Clínico San Carlos Hospital, Madrid, Spain

## Abstract

**Background:**

Mandibular third molar (MTM) extraction is one of the most frequently performed surgeries in the oral cavity. Establishing the level of surgical difficulty pre-operatively is an essential step to ensure correct treatment planning. In Spain, MTM extraction - especially in cases presenting greater difficulty - is normally performed by doctors specializing in oral and maxillofacial surgery, or by dentists with postgraduate qualifications in oral surgery. The present work set out to analyze the extent to which perceptions of surgical difficulty of the said intervention vary in relation to professional training.

**Material and Methods:**

This cross-sectional, descriptive, observational study took the form of a survey. Using a visual analog scale (VAS), participants evaluated both the perceived difficulty of 30 cases of MTM extraction described by means of digital panoramic radiographs and the perceived difficulty deriving from a series of factors conditioning MTM extraction. The results underwent statistical analysis with SPSS Statistics 28.0 software. Non-parametric tests (Mann Whitney test for independent samples and the Kruskal-Wallis test) were applied.

**Results:**

A total of 213 surveys were available for analysis. Both groups awarded the greatest importance to clinical experience, followed by anatomical and radiographic factors, root morphology obtaining the highest score among anatomical factors (9.01±1.42), while proximity of the MTM to the inferior alveolar nerve was regarded as the least important anatomical factor (8.11±2.54). Significant differences were only found for patient age, whereby maxillofacial surgeons awarded this factor more importance than dentists.

**Conclusions:**

The different training received by dentists specialized in oral surgery and maxillofacial surgeons did not influence either perceptions of surgical difficulty of MTM extraction, or opinions as to the factors influencing surgical difficulty.

** Key words:**Third molar, mandibular, extraction, perceived difficulty, professional training, maxillofacial surgeon.

## Introduction

Mandibular third molar (MTM) extraction is one of the most frequently performed surgeries in the oral cavity (1). It can be difficult, laborious, detailed and demands the right, carefully controlled protocols and techniques.

Given the multiple situations and positions that impacted MTMs can present, it is impossible to establish or describe a single type of intervention that encompasses all eventualities. For this reason, various authors have set out to determine classification systems and scales of surgical difficulty aimed at assisting the surgeon’s approach to the individual case of impacted MTM. The most widely used classifications are those by Winter (2), Pell and Gregory (3), and Pederson (4).

In addition, some dental professionals have created their own difficulty scales based on different ranges of variables (5-9). Initially, radiographic variables were analyzed in orthopantomographs such as the size and shape of the tooth crown, the number, size and curvature of the roots, the impaction’s position and situation, the presence or absence of periodontal ligament, or relationships with adjacent structures (2-5,10,11). Later, other variables were included as factors influencing surgical difficulty, some related to the patient (age, sex, ethnicity, body mass index, mouth opening, etc.), others related to operative conditions (need for flap raising, for ostectomy, for odontosection, the surgeon’s experience, etc.) (5,12-16).

In all cases, establishing the level of surgical difficulty preoperatively is essential for correct treatment planning. This will make it possible to prepare the right materials, decide the point of surgical access, determine the appropriate technique, the type of anesthesia, and to determine whether or not the operative’s experience and capabilities match the extraction to be performed (17). In this context, various authors have assessed dentists’ capacity for predicting surgical difficulty, obtaining widely differing results (12,18,19).

In Spain, MTM extraction - especially in cases presenting greater difficulty - is normally performed by doctors specializing in oral and maxillofacial surgery, or by dentists with postgraduate qualifications in oral surgery. But these two groups undergo different training in surgical procedures in quite different settings.

The present work set out to analyze the extent to which perceptions of surgical difficulty of the said intervention vary in relation to professional training. To the authors' knowledge, no previously published studies have compared the perception of surgical difficulty between oral and maxillofacial surgeons and dentists specialized in oral surgery.

We felt it would be useful and relevant to compare (by means of a survey) two groups, the first comprising oral and maxillofacial surgeons, the second dentists specializing in oral surgery, in terms of both the perceived levels of difficulty of a variety of cases of MTM extraction, and the perceived influence of a series of factors (patient-related, anatomical and radiographic, and operative factors) on difficulty.

## Material and Methods

- Study design, survey validation, and sample size

This cross-sectional, descriptive, observational study took the form of a survey. The survey was formulated with EUSurvey software, the European Commission’s official tool for conducting surveys.

The survey was designed by a team of oral surgery specialists and was informed by the available literature addressing the difficulty levels and factors influencing MTM extraction. Before delivery to participants, the team presented the survey to four experts in oral surgery who did not participate in the study. They were asked to analyze the survey questions in terms of clarity and ease of comprehension. Their corrections and suggestions were introduced, giving the survey its final form (20).

Lastly, the Intraclass Correlation Coefficient (ICC) and Cronbach's alpha were calculated to evaluate variability in repetition of the survey, and reliability of the measurement scale, respectively.

Invitations were sent to potential participants, explaining the objectives and reasons for the survey and its approximate duration. The survey was made available online to those professionals willing to participate.

The participants had received varying types of training and acquired different levels of experience. They included either dental graduates who had completed or were completing a postgraduate degree in oral surgery or maxillofacial surgeons who had, or had not, completed their training hospital internship.

A link to the survey was disseminated by e-mail and via Whatsapp to current students, former students, teaching staff, work colleagues, and research colleagues at a range of Higher Education Centres and hospitals in Madrid (Spain).

Before completing the survey, participants gave their informed consent to take part in the study. In turn, they were provided with a guarantee of anonymity and data privacy.

The survey consisted firstly of a series of items related to the participants’ demographic and academic data (age, sex, academic situation, year of graduation, type of postgraduate degree in oral surgery, year of completion of postgraduate degree, year of specialization in maxillofacial surgery in the case of physicians).

Thereafter, using visual analogue scales (VAS), the participants assessed the importance of a series of patient-related factors (sex, age, ethnicity, mouth opening, body mass index), anatomical and radiographic factors (root morphology, root curvature, lower third molar position, lower third molar situation, depth of impaction, impaction of lower third molar in the ascending ramus, proximity of the lower third molar to the inferior alveolar nerve), and operative factors (anesthetic technique: local vs. general anesthesia; need for flap lifting; need for ostectomy; need for odontosection; clinician’s experience), which could influence the surgical difficulty of lower third molar extraction. On the scale, “0” represented “minimum influence” on surgical difficulty and “10” represented “maximum influence.”

Lastly, participants assessed (using VAS) the levels of difficulty of 30 cases of MTM extraction described by high quality digital panoramic radiographs, all of which showed at least one MTM. The radiographs were selected from those pertaining to patients treated on the Master’s Program in Oral Surgery and Implantology at the Complutense University of Madrid, who gave their consent for their radiographs to be used anonymously in the study.

The sample size was determined according to a previous, similar study (12) and one of the main variables: age. A preliminary sample size calculation was made using specialized software (G*Power 3.1.9.4). The calculation revealed a total sample size of 11 participants per group with an effect size of 21,1 at 0.8 power and a significance level of 0.05.

- Study approval

The study was approved by the Committee for Ethics in Research at the San Carlos Hospital, Madrid, Spain (C.I. 22/135-E), and followed the ethical guidelines established in the Declaration of Helsinki by the World Medical Association. The study was conducted following STROBE (Strengthening the Reporting of Observational Studies in Epidemiology) guidelines (21).

- Data collection and statistical analysis

The results of the survey were entered and stored on a EUSurvey spreadsheet and later underwent statistical analysis with SPSS* Statistics 28.0 software (SPSS® inc, Chicago IL, USA).

Statistical analysis was conducted at the Data Processing Center of the University. Firstly, a descriptive study of frequencies was performed, calculating means, median values, standard deviations, and ranges. Secondly, data were analyzed with inferential statistics with a 95% Confidence Interval, and so a significance level of *p*<0.05.

Applying the Kolmogorov-Smirnov test, it was found that data did not display normal distribution, and so non-parametric tests were used: the Mann Whitney test for independent samples (two comparative groups) and the Kruskal-Wallis test (more than two comparative groups). Whenever the Kruskal-Wallis test indicated significant differences, paired comparisons were made with Bonferroni corrections.

## Results

- Subject characteristics

The survey was sent to 385 people, of whom 214 (55.58%) completed the survey online. One participant later refused to provide informed consent to take part, so 213 (55.32%) surveys were available for analysis. Distribution of the sample in terms of age, sex, and professional training is shown in Table 1.

Most of the participants (84.51%) were dentists who were attending or had completed some post-graduate course in oral surgery. The other group (15.49%) consisted of maxillofacial surgeons (doctors specialized in maxillofacial surgery) who had completed or were completing hospital internships in the specialization.

Dentists were asked to state the time passed since completing postgraduate surgical training (or whether they were currently undergoing training). They were also asked which education center they had attended or were attending. The responses named seven different centers, including public Universities, private Universities and other private educational centers in the Madrid area, the Complutense University of Madrid being the most frequently cited.

In the same way, maxillofacial surgeons were asked how long it had been since completing their internship or whether they were currently doing an internship, and at which hospital, the San Carlos Clinical Hospital Madrid being the most frequently cited.

- Survey validation

The survey was repeated one week later by a large and representative sample of participants (16.43%) in order to evaluate the variability of the survey. The ICC was calculated for every participant determining excellent concordance in all cases (values >0.8), confirming the survey’s validity. In addition, Cronbach's alpha was calculated to measure the reliability of the measurement scale, obtaining a value of 0.83, which guaranteed its reliability.

To provide a detailed and comprehensible analysis of the results, three comparative groups were established (Table 2).

**Table 1:**
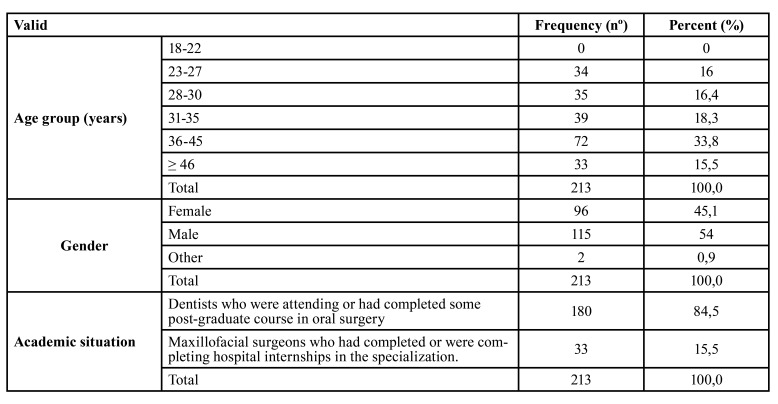
Sample distribution by age, sex, and academic situation.

**Table 2:**
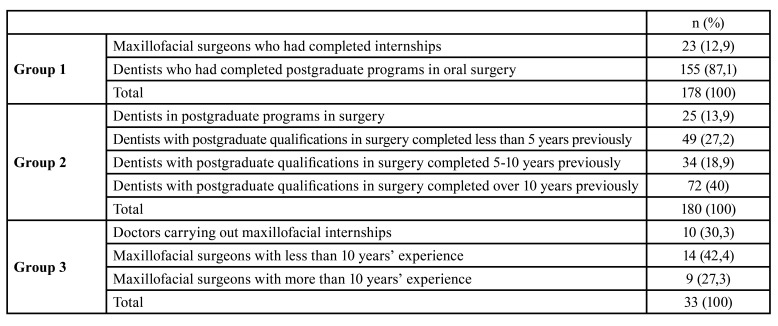
Comparative groups.

- GROUP 1 (Maxillofacial surgeons who had completed internships/Dentists who had completed postgraduate programs in oral surgery)

Results obtained from maxillofacial surgeons and from dentists with postgraduate surgical training were compared with the Mann-Whitney U test.

Both groups awarded the greatest importance to clinical experience, followed by anatomical and radiographic factors, root morphology obtaining the highest score among anatomical factors (9.01±1.42), while proximity of the MTM to the inferior alveolar nerve was regarded as the least important anatomical factor (8.11±2.54) (Table 3).

**Table 3:**
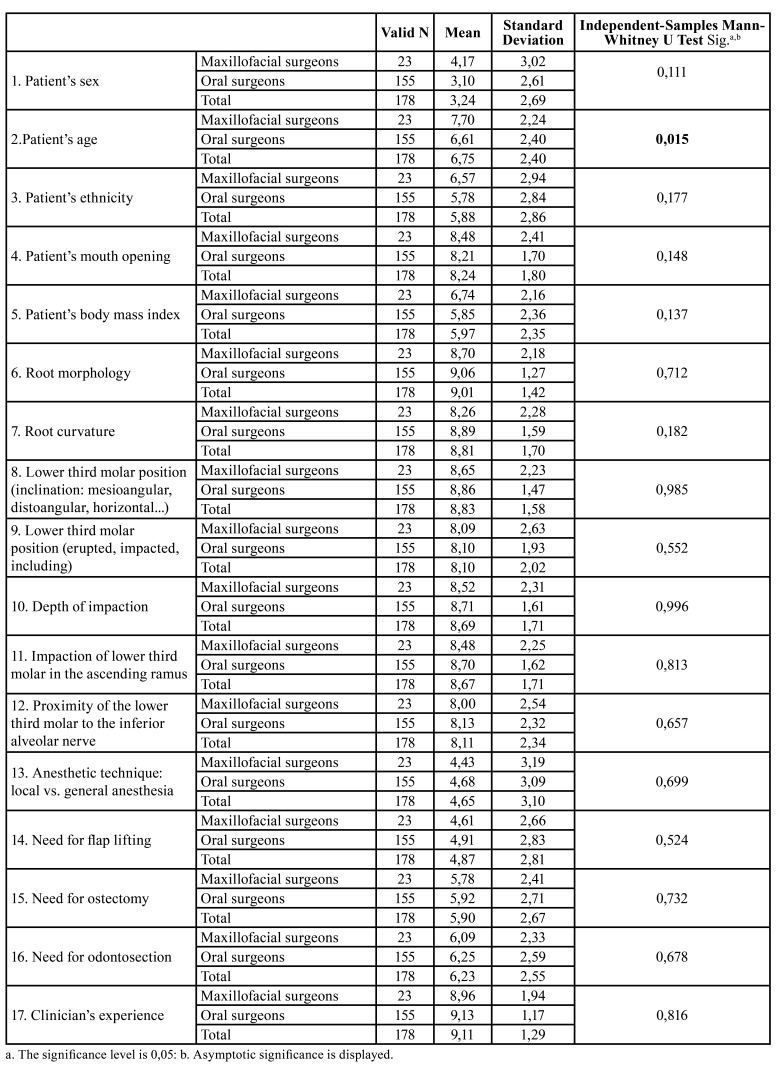
Values given by physicians specializing in oral and maxillofacial surgery, or by dentists with postgraduate degrees in oral surgery, to the various surgical difficulty factors analyzed.

Comparing differences in perceptions of the influence of the factors listed above on surgical difficulty, significant differences (*p*<0.05) between the two groups were only found for patient age, whereby maxillofacial surgeons awarded this factor more importance than dentists. Maxillofacial surgeons generally placed greater importance on demographic and patient-related factors than dentists although these differences did not reach statistical significance. Dentists gave higher scores to anatomical, radiographic, and operative factors than maxillofacial surgeons, but again without statistically significant difference.

Both groups considered the patient’s sex a factor of little influence (3.24±2.69) and clinical experience the most influential (9.11±1.29). Mouth opening (8.24±1.70) and age (6.75±2.4) were the most important patient-related factors for both groups. Among operative factors, after experience, the need for odontosection (6.23±2.55) was considered the second most influential factor.

**Figure 1:**
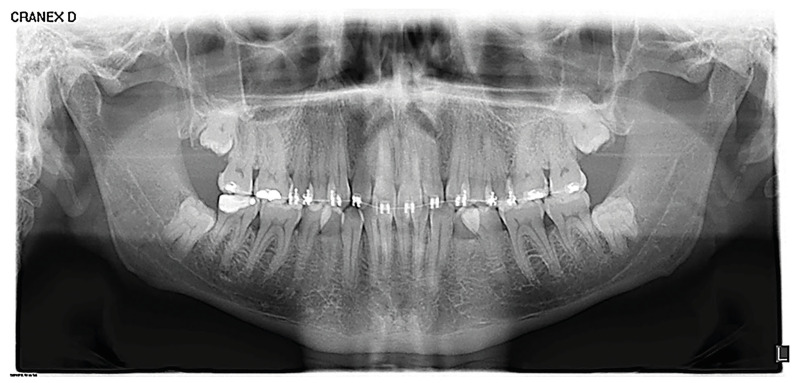
Panoramic radiograph number 25 used in the survey for evaluation of MTM 38.

**Figure 2:**
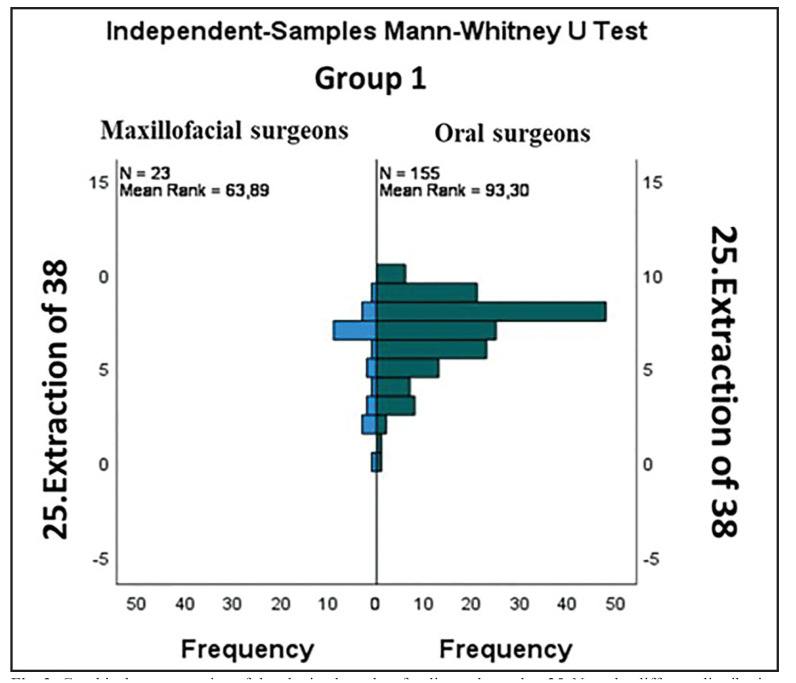
Graphical representation of the obtained results of radiograph number 25. Note the different distribution by groups, where dentists with training in oral surgery gave higher scores than maxillofacial surgeons.

Comparing the perception of difficulty of the 30 cases of impacted MTM included in the survey with the Mann Whitney U test, higher scores were given by dentists specialized in oral surgery than maxillofacial surgeons in all cases, even though statistically significant differences (*p*<0.05) were only found in four cases (Fig. 1, Fig. 2).

- GROUP 2 (Dentists in postgraduate programs in surgery/ Dentists with postgraduate qualifications in surgery completed less than 5 years previously/ Dentists with postgraduate qualifications in surgery completed 5-10 years previously / Dentists with postgraduate qualifications in surgery completed over 10 years previously)

Survey scores were analyzed for dentists with specialized training in oral surgery in relation to years of clinical experience, differentiating between those still in training, those who had completed training less than 5 years previously, 5-10 years previously, and over 10 years previously.

All considered that, after clinical experience (9.16 +/- 1.13), anatomical and radiographic factors had the most influence on surgical difficulty. Of these, root morphology (9.07± 1.25) and curvature (8.92±1.58) and the position of the MTM (8.91±1.41) were regarded as the most influential.

**Figure 3:**
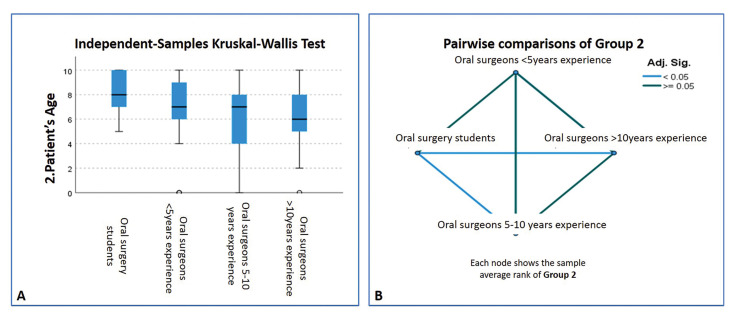
Assessment of the factor "Patient’s age" within Group 2. (A) Boxplot. (B) Graphical representation of the pairwise comparison. Note that there are statistically significant differences between the dentists in postgraduate programs in oral surgery and both, those dentists with postgraduate qualifications in surgery completed 5-10 year previously and dentists with postgraduate qualifications in surgery completed over 10 years previously.

Comparing the subgroups in terms of the importance of different factors, statistically significant differences (*p*<0.05) were only found for three of the 17 factors (age, body mass index, and MTM position), whereby these were allotted more importance by professionals with less experience (Table 4, Fig. 3).

**Table 4:**
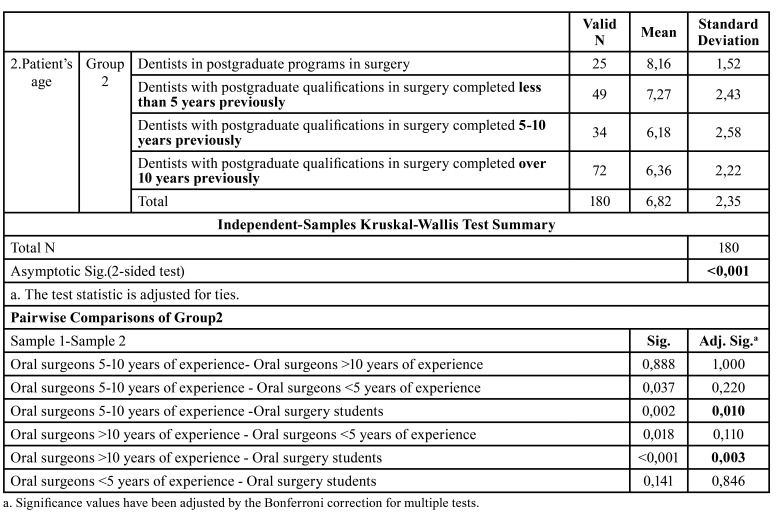
Values ​​given by dentists in postgraduate programs in surgery/ dentists with postgraduate qualifications in surgery completed less than 5 years previously/ dentists with postgraduate qualifications in surgery completed 5-10 years previously / dentists with postgraduate qualifications in surgery completed over 10 years previously to the factor “Patient´s age”. Kruskal-Wallis test and pairwise comparison.

The patient’s sex (3.15±2.6) was considered the least important factor. Patient-related factors were considered more important than operative factors, mouth opening (8.22±1.77) being regarded as the most influential, followed by age (6.85±2.35). Need for odontosection (6.39± 2.56) was seen as the most influential operative factor, after clinical experience.

Comparing the difficulty scores awarded to the 30 cases of impacted MTM by means of the Kruskal Wallis test, significant differences (*p*<0.05) were found in only one case.

- GROUP 3 (Doctors carrying out maxillofacial internships / Maxillofacial surgeons with less than 10 years’ experience / Maxillofacial surgeons with more than 10 years’ experience)

Kruskal Wallis test was applied for comparing these three groups.

Regardless of subgroup/experience, all maxillofacial surgeons considered clinical experience the most important factor, followed by anatomical and radiographic factors.

**Figure 4:**
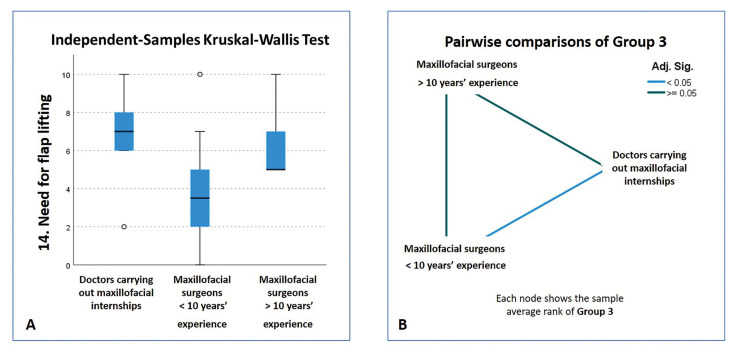
Assessment of the factor "Need for flap lifting" within Group 3. (A) Boxplot. (B) Graphical representation of the pairwise comparison. Note that there is a difference between doctors carrying out maxillofacial internship and maxillofacial surgeons with less than 10 years` experience.

Analyzing the importance placed on the different factors, only three of the 17 factors obtained significant differences between subgroups, all of them operative (need for flap raising, need for ostectomy, need for odontosection), whereby maxillofacial surgeons with less than 10 years’ experience awarded them lower scores (Fig. 4).

This group placed greater importance on patient-related factors (with the exception of patient sex) than operative factors, in particular, mouth opening, followed by age, body mass index, and ethnicity.

No significant differences between subgroups were found in the difficulty values awarded to the 30 cases. Nevertheless, those engaged in internships gave higher scores.

## Discussion

Determining the potential difficulty of MTM extraction surgery pre-operatively is essential for correct surgical planning and helps minimize risk and avoid possible intra- and post-operative complications. In Spain, this intervention may be performed by a physician specialized in maxillofacial surgery or by a dentist, so a comparison of how these two groups perceive the difficulty of MTM extraction and the factors influencing levels of difficulty is both revealing and clinically relevant.

Several researchers have assessed dentists’ ability to predict surgical difficulty with widely differing results (12,18,19,22-25). But in the present work, we set out to investigate perceived difficulty rather than predicted difficulty (which would involve actual extractions afterwards).

The main objective was to assess whether perceived difficulty varies in relation to the surgical training received, either as a ‘MIR’ (‘Médico Interno Residente’ - Medical Doctor’s Internship at a hospital), which is how doctors receive training in maxillofacial surgery in Spain, or as a post-graduate specialization in oral surgery, which is how dentists are trained in oral surgery in Spain.

Assessing the levels of difficulty of the 30 selected cases of MTM, lower VAS scores were given by maxillofacial surgeons, although differences in comparison with dentists did not reach statistically significant difference. Barreiro *et al*. (18) compared the capacity to predict surgical difficulty of 10 dentists specialized in oral surgery, two general dentists working in primary care, and two maxillofacial surgeons, finding a greater predictive capacity among the specialized dentists and the maxillofacial surgeons compared with the general dentists. This could be due to better training in selecting the right technique and approach to MTM extraction and in the factors that affect the complexity of the procedure. Nevertheless, the authors observed a tendency to underestimate the level of difficulty among all the professionals (general dentists, dentists specialized in oral surgery, and maxillofacial surgeons), which was particularly accentuated among the maxillofacial surgeons.

In the present study, the scores allotted to the cases of impacted MTM described by means of panoramic radiographs were higher among those professionals with less experience in both groups, although differences did not reach statistically significant difference. This observation concurs with data supplied by Ferrús-Torres *et al*. (23) and Pippi (24), who reported that they found clinical experience to be an important factor contributing to the capacity to predict surgical difficulty, whereby professionals with less experience tend to assess the procedure as more difficult than those with more experience. But Susarla *et al*. (12,22) did not find any relation between years of experience and predictive capacity.

The present work also set out to determine which demographic and patient-related, anatomical/radiographic, and operative variables were thought to have more or less influence on surgical difficulty. Susarla and Dodson reported that professionals considered anatomical factors more important, followed by operative factors, and lastly patient-related and demographic variables (12). In contrast, in the present work, while anatomical/radiographic factors were thought the most influential, the results indicate that both dentists specialized in oral surgery and maxillofacial surgeons place greater importance on patient-related factors than on operative factors, with the exception of clinical experience, the factor that both groups considered the most influential. These results concur with findings published by Akadiri *et al*. and Susarla *et al*. (5,12).

The high assessment awarded to these other factors (patient-related variables) appears to be crucial, as, according to Susarla and Dodson (12,22), it is precisely the fact of not considering these factors - whether through unawareness or failure to pay them due attention - that leads to an inadequate estimation of surgical difficulty. It has been shown that age (due to higher bone density, and greater degree of ankilosis among older patients), race (due to higher bone density and higher frequency of hypercementosis (bulbous roots) among black patients) have direct influences on surgical difficulty (26-28). So, it is crucial to stress the importance of these factors when training students and interns in the highly variable procedures and difficulties involved in MTM extraction.

In agreement with Akadiri *et al*. and Susarla *et al*. (5,12), all the professionals who participated in the present work considered that the most important factors (after clinical experience) were anatomical and radiographic variables, in particular, root morphology and curvature. Both groups considered the patient’s sex the least influential factor, a finding that agrees with previous studies (22,24,29,30).

This study suffered some limitations, namely the fact that the participants were restricted to a population of professionals all trained in and around Madrid, as well as the unequal numbers forming each group and subgroup.

## Conclusions

Perceptions of surgical difficulty of MTM or the factors influencing difficulty do not vary in relation to professional training. Furthermore, years of clinical experience did not affect assessments of surgical difficulty significantly, although those with longer experience tended to give lower values for difficulty than less experienced professionals.
